# Epidemiologic Pictures of Kawasaki disease in Shanghai from 1998 through 2002

**DOI:** 10.2188/jea.16.9

**Published:** 2005-12-20

**Authors:** Guo-Ying Huang, Xiao-Jing Ma, Min Huang, Shu-Bao Chen, Mei-Rong Huang, Yong-Hao Gui, Shou-Bao Ning, Tuo-Hong Zhang, Zhong-Dong Du, Hiroshi Yanagawa, Tomisaku Kawasaki

**Affiliations:** 1 Children’s Hospital of Fudan University, Department of Pediatrics of Shanghai Medical College.; 2 Children’s Hospital of Jiaotong University.; 3 Shanghai Children’s Medical Center of Xinhua Hospital.; 4 School of Public Health, Beijing University.; 5 Beijing Children’s Hospital.; 6 Saitama Prefectural University.; 7 Japan Kawasaki Disease Research Center.

**Keywords:** Mucocutaneous Lymph Node Syndrome, Epidemiology, Incidence, Cardiovascular Diseases, China

## Abstract

**BACKGROUND:**

Epidemiologic features of Kawasaki disease in China is still not clear.

**METHODS:**

A questionnaire form and diagnostic guidelines for Kawasaki disease were sent to hospitals in Shanghai, which provided with pediatric medical care. All patients with Kawasaki disease diagnosed during January 1998 through December 2002 were recruited in this study.

**RESULTS:**

A total of 768 patients with Kawasaki disease were reported. The incidence rates of Kawasaki disease for each year were 16.79 (1998), 25.65 (1999), 28.16 (2000), 28.05 (2001), and 36.76 (2002) per 100,000 children under 5 years of age. The male/female ratio was 1.83:1. The age at onset ranged from 1 month to 18.8 years (median: 1.8 years). The disease occurred more frequently in spring and summer. Fever was the most common clinical symptom, followed by oral changes, extremities desquamate, rash, conjunctive congestion, lymphadenopathy, extremities swelling, and crissum desquamate. Cardiac abnormalities were found in 24.3% of patients. The most common cardiac abnormality was coronary artery lesions including dilatation (68%) and aneurysm (10%). The case-fatality rate at acute stage of the disease was 0.26%. A second onset of the disease occurred in 1.82% of patients.

**CONCLUSIONS:**

The incidence rate of Kawasaki disease in Shanghai is lower than that reported in Japan, but higher than those in western countries. The increasing trend in incidence, sex distribution and cardiac abnormalities are similar to those in previous reports. The seasonal distribution is similar to the report from Beijing and different from other reports.

Kawasaki disease (KD) is a disease of unknown etiology affecting most frequently infants and young children under 5 years of age. The disease has attracted more and more attention since it was reported by Dr. Kawasaki in 1967.^1^ In recent years, it has been reported as the leading cause of acquired heart disease of childhood in Japan and North America.^2^^,^^3^ Unfortunately, the cause of KD remains unknown although many advances have been achieved in treatment and research on etiology.^4^^,^^5^ The reported mean annual incidence rate of KD varies in different countries and districts, which was 90 to 112 per 100,000 in Japan,^6^^-^^9^ 8.0 to 47.7 per 100,000 in the United States,^10^^-^^12^ and 3.6 to 3.7 per 100,000 in the United kingdom and Australia^13^^,^^14^ for children under 5 years of age. The disease is supposed to be more common in the Asian race.^15^ However, several reports showed that the incidence rate of KD was 2.2 per 100,000 in Northeast Thailand.^16^ 54.9 per 100,000 in Taiwan,^17^ and 25.4 per 100,000 in Hong Kong for children under 5 years of age.^18^ Given these, together with a recent report showing the incidence rate of KD in 18.2 to 30.6 per 100,000 for children under 5 years of age in Beijing, one of the developed district in China,^19^ we hypothesis that KD may be more common in Japanese children rather than in other Asian children.

This study was to display the epidemiologic picture of KD in Shanghai, the developed area with the largest population in the city in China, based on the retrospective survey conducted from 1998 through 2002.

## METHODS

### Case Ascertainment

A survey questionnaire form and diagnostic guidelines of KD (The 5th revised edition issued by the Japan Kawasaki Disease Research Committee in 2002) were sent to 50 hospitals that provide with pediatric care in Shanghai including its suburbs with a population of 16 million. The questionnaire form and the diagnostic guidelines were provided in Chinese and were the same as those used in the nationwide epidemiologic surveys in Japan.^6^^-^^9^ Patients with KD were identified by the discharge diagnosis code in medical records. Pediatricians were asked to review the medical records and report all patients with KD diagnosed during the 5-year period from January 1998 through December 2002. All pediatricians who participated in the survey were either pediatric cardiologists or senior pediatricians. Two senior pediatricians were designated to ensure investigator compliance with the study protocol. Questions in the questionnaire form consist of: date of diagnosis, date of birth, sex, address of inhabitance, date of onset of KD, days from onset of illness to hospital visit, clinical findings, cardiac manifestations, treatment, and prognosis. The cardiac abnormalities were defined as presence of dilatation, aneurysm, stenosis or occlusion of coronary artery, myocardial infarction, valvular lesions or pericardial effusion within 1 month after the onset of KD evaluated by echocardiography or coronary angiography.

### Definition and Recruited Criteria

Cases were included in the study if the patients had at least five of the following six clinical signs: (1) fever persisting 5 days or more (inclusive of those cases in whom the fever has subsided before the 5th day in response to therapy); (2) bilateral conjunctival congestion; (3) changes of lips and oral cavity: reddening of lips, strawberry tongue, diffuse injection of oral and pharyngeal mucosa; (4) polymorphous exanthema; (5) changes of peripheral extremities: reddening of palms and soles, indurative edema at initial stage, or membranous desquamation from fingertips at convalescent stage; and (6) acute nonpurulent cervical lymphadenopathy. Patients were also included if they had four of the above signs with coronary abnormalities documented by two-dimensional echocardiography or coronary angiography. Cases were excluded if the questionnaire form was not completed correctly, or if they were not satisfied for the diagnostic guidelines of KD. The definition of coronary abnormalities by echocardiography was based on the criteria established by the Kawasaki Disease Research Committee in Japan.^20^

### Analysis of Risk Factors of Coronary Artery Lesions

The potential risk factors of coronary artery lesions were analyzed, which included age at onset, sex, clinical symptoms and signs, erythrocyte sedimentary rate, maximum blood platelet count, C-reactive protein, serum albumin level, lesions in other systems, administration of intravenous gamma-globulin, administration of corticosteroid and recurrence of KD.

### Statistics

Parametric data were expressed as median or mean and standard deviation (SD). The incidence rate of KD was calculated by dividing the number of positive cases by the population data obtained from the Census Office of Shanghai Municipality. Nonparametric analysis was performed using the Pearson chi square test or Fisher’s exact test. Difference of means was compared by t tests or one-way analysis of variance. Logistic regression analysis was carried out to analyze the risk factors for coronary artery lesions. The confidence interval was calculated as appropriate. SPSS^®^ 11.0 (SPSS Inc., Chicago, IL) for Windows was used for the analysis. A probability less than 0.05 was considered as statistically significant.

## RESULTS

### Incidence Rate and Epidemic Pictures

All the 50 hospitals, to which the questionnaires were sent, responded to the survey. A total of 784 potential patients were reported. Of these, 18 cases (2.3%) that did not satisfy our recruited criteria were excluded. The remaining 768 patients who satisfied the inclusion criteria were enrolled as study subjects. They included 737 patients (96.0%) who satisfied 5 or 6 of the 6 clinical signs and 31 patients (4.0%) who had only 4 of the 6 signs plus abnormal coronary arteries identified by echocardiography.

**Figure 1.**
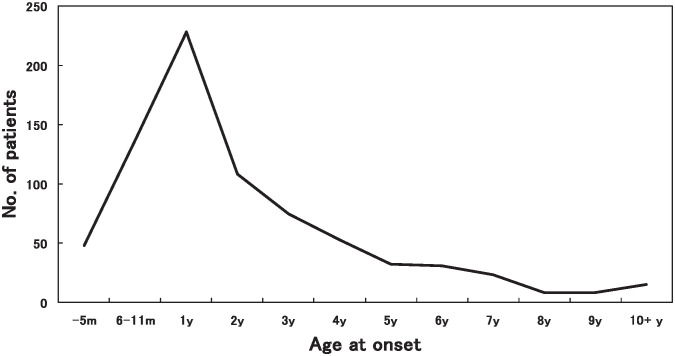
Distribution of age at onset of Kawasaki disease in Shanghai, China, 1998-2002.

There were 497 boys and 271 girls, with a male/female ratio of 1.83:1. Their ages at onset ranged from 1 month to 18.8 years (median: 1.8 years). Patients under 1 year and 5 years of age accounted for 26.9% and 85.4%, respectively. The highest incidence rate of KD was observed in 9.6 month of age (Figure 1).

The incidence rate of KD varied significantly from 1998 through 2002. Based on population census data in Shanghai, the average annual incidence of KD tended to increase by years (Table 1).

**Table 1.** The number of patients and incidence rate of Kawasaki disease by calendar year and sex in Shanghai, China.

Calendar year	Both sexes		Males		Females	
		
	No. of patients	Incidence rate*	No. of patients	Incidence rate*	No. of patients	Incidence rate*
1998	88	16.8	51	18.6	37	14.8
1999	131	26.7	97	36.3	34	14.0
2000	138	28.2	91	35.4	47	20.2
2001	131	28.1	80	32.9	51	21.8
2002	166	36.8	114	48.8	52	23.9

p^†^	<0.001		<0.001		<0.05	

*: Incidence rate per 100,000 children under 5 years of age

†: Chi-square tests

**Figure 2.**
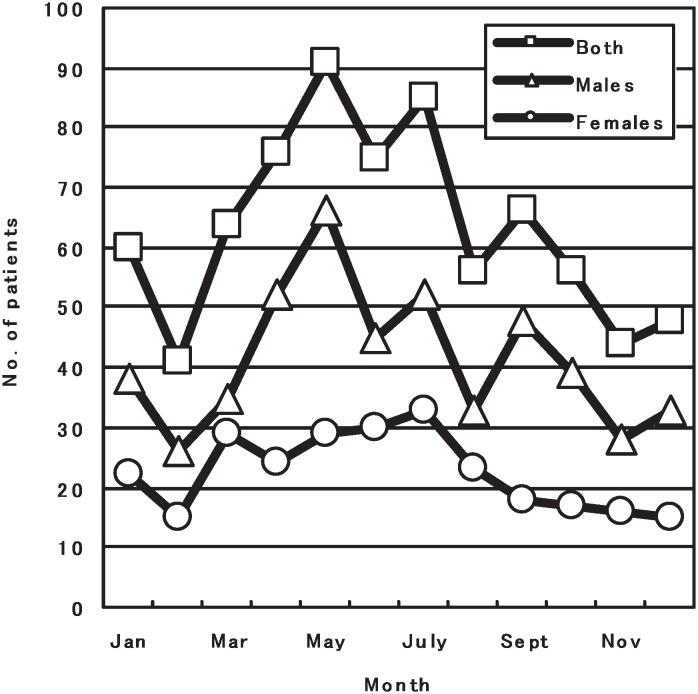
The number of patients with Kawasaki disease by month, Shanghai, China, 1998-2002.

Seasonal variation was observed. In general, KD was more prevalent in spring and summer. This was more obvious in girls, and the increase of the patients started in March, and the highest peak was in July. However, the highest peak for boys was in May, and there was another small peak in September. The lowest occurrence was in February (Figure 2).

Comparison of ages at various months revealed that patients in December and January were older (average±SD: 3.7±3.8 and 3.8±3.1 years) than those who had the disease in other months of the year (from 2.0±1.6 years in October to 3.1±3.2 years in July; F=3.194, p=0.000).

### Clinical Manifestations

The duration from the onset of the first symptom through diagnosis ranged from 1 to 60 days (average: 10 days). Sixty-two percent of the patients were diagnosed within 10 days from the onset. Fever persisting for 5 days or longer was the most common symptom, followed by changes of lips and oral cavity, membranous desquamation from fingertips, rashes, bilateral conjunctive congestion, cervical lymphadenopathy, and indurative edema at extremities. Interestingly, crissum (perianal region) desquamate was found in 45.2% of the patients in this study, which was seldom mentioned in the previous reports (Table 2).

**Table 2.** Clinical manifestations of the 768 patients with Kawasaki disease, Shanghai, China.

Clinical symptoms	No.	(%)
Fever	763	(99.3)
Oral and lip changes	641	(83.5)
Extremities desquamate	637	(82.9)
Rashes	622	(81.0)
Conjunctive congestion	602	(78.4)
Lymphadenopathy	532	(69.3)
Extremities swelling	369	(48.1)
Crissum desquamate	347	(45.2)

### Intravenous Gamma-globulin Administration

Infusion of intravenous gamma-globulin (Human Freeze-Dried Low PH Intravenous Gamma Globulin; Biochemical Product Institute of Ministry of Public Health, Chengdu, China) was administered to 550 patients (71.8%) in 1 to 24 days after the onset of the disease (median: 7 days). The dosage was 400 to 500 mg/kg/day for 5 consecutive days in 145 patients (26.4%), 1000 mg/kg once in 182 patients (33.1%), 2000 mg/kg once in 160 patients (29.1%), and in other ways in 63 patients (11.4%), respectively. However, infusion of intravenous gamma-globulin was administered to 446 out of 550 patients within 10 days after onset of the disease.

**Figure 3.**
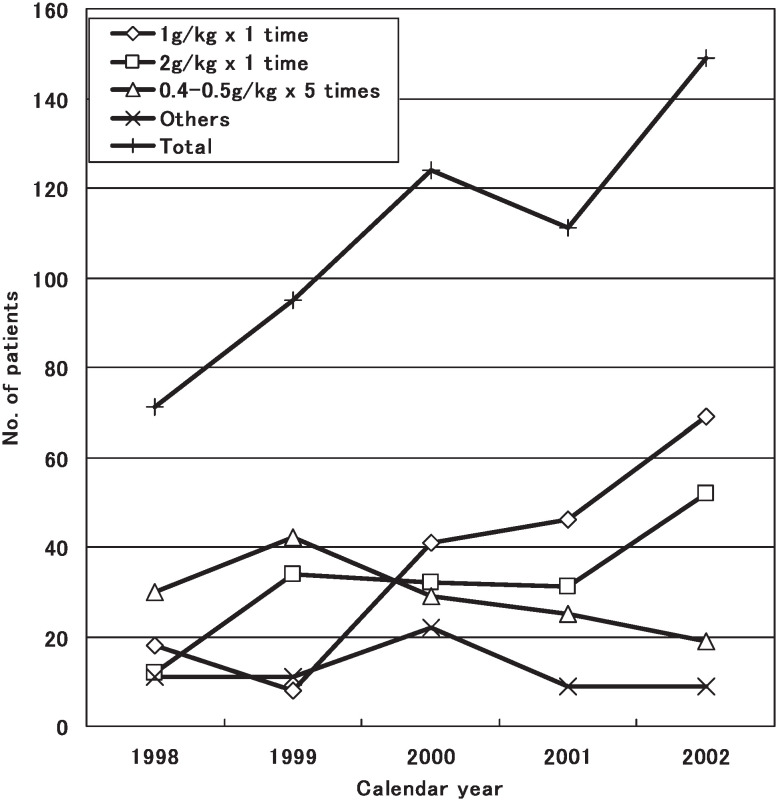
Dosage of intravenous gamma-globulin for Kawasaki disease by year, Shanghai, China, 1998-2002.

It was shown that more patients received the gamma-globulin treatment in the later years, and the dosage of 1000 mg/kg once or 2000 mg/kg once had been used more often than other dosage since 2000 (Figure 3). Out of 550 patients, only 1 case (0.18%) had adverse effect due to the administration of gamma-globulin who had transient urticaria.

### Cardiac Abnormalities and Its Risk Factors

Data on cardiac findings based on echocardiography were available in 731 patients. Of these, 186 (24.3%) had cardiac abnormalities occurred on initial 2 days to 8.7 months (average±SD: 15.0±22.1 days). The proportion of cardiac abnormalities was 27.1% in boys and 22.4% in girls, respectively (p=0.161). The cardiac abnormalities were diagnosed in 71.0% of patients within 15 days after onset and 29.0% after 15 days. The highest occurrence rate of cardiac abnormalities was observed in 8.4 month of age with 37.1% in patients under 1 year of age. The most common cardiac abnormality was dilatation of coronary arteries, which accounted for 68%, followed by coronary aneurysm (10%), valve lesions (9%), pericardial effusion (7%), and heart failure (1%). The occurrence rate of the coronary artery lesions (CAL) including dilatation and aneurysm was 19.8% (145/732). Giant coronary aneurysm accounted for 2.1% (3/145) among CAL and 15.8% of coronary aneurysm.

It was shown that patients with CAL were different from those without CAL in age at onset (2.19±2.06 years vs 2.80±2.50 years), the maximum blood platelet count (569.1×10^9^±191.8×10^9^/L vs 518.4×10^9^±174.9×10^9^/L), serum albumin level (34.3±5.78 g/L vs 36.6±6.74 g/L). It was also shown that administration of intravenous gamma-globulin and administration of corticosteroid were related to CAL. In our data, 41 patients received corticosteroid treatment and 17 had CAL with the proportion of 41.5%, which was significantly higher than other patients (p=0.001). However, logistic regression analysis showed that the risk factors of CAL were hypoalbuminemia (odds ratio = 0.99, 95% confidence interval = 0.99-1.0), and administration of corticosteroid (odds ratio = 1.22, confidence interval = 1.03-1.43).

**Figure 4.**
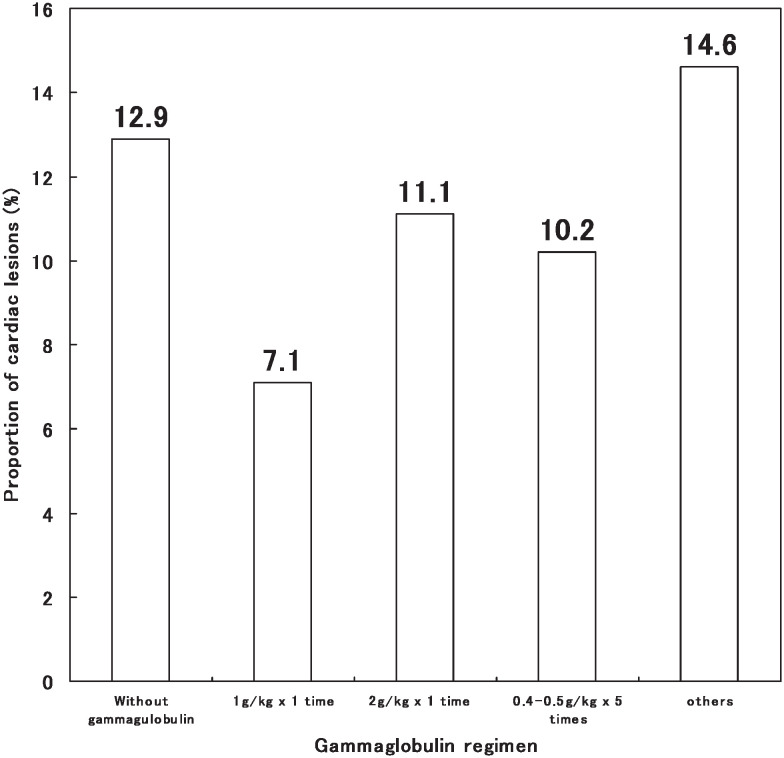
Proportion of cardiac lesions due to Kawasaki disease by intravenous gamma-globulin regimen, Shanghai, China, 1998-2002.

Interestingly, it was shown that occurrence of CAL tended to be less common in the patients who received treatment of intravenous gamma globulin within 10 days of onset, especially in the dosage of 1g/kg×once although the Pearson chi square test did not display the statistic significant differences (p=0.575) (Figure 4).

### Prognosis

There were 2 deaths during the acute and subacute stages of the disease with the case-fatality rate of 0.26%. One died of rupture of coronary aneurysm on day 14 of onset and the other died of congestive heart failure on day 17 of onset. Fourteen patients (1.82%) had a second onset of the disease in 1-35 months (median: 7 months) after the first onset. No specific factors associated with the recurrence were observed in this study.

## DISCUSSION

This study reported the frequency and epidemiologic features of Kawasaki disease in Shanghai, one of the developed areas with the largest population in the city in China. The annual incidence rate of KD in our study was 16.6-36.8 per 100,000 for children under 5 years old from 1998 through 2002, which was much lower than that reported in Japan, which were about 5-fold higher than the incidence in our study.^6^^-^^9^ Because the diagnostic criteria and survey protocol were same as those used in the nationwide survey in Japan, and all the hospitals which provided with pediatric care participated in this study, there would be less chance to miss a large number of patients as the incidences reported from Japan. Moreover, the incidence rate of KD in our study is similar to that in Beijing and Hong Kong, other developed areas with large population in the city in China. The incidence rate of KD in Beijing was 18.2-30.6 per 100,000 for children under 5 years from 1995 through 1999.^19^ In an earlier report, a descriptive account of patients at one hospital in Hong Kong from 1989 through 1994 had shown an incidence of 25.4 per 100,000 for children under 5 years old.^18^ A recent report showed that the incidence of KD was 54.9 per 100,000 for children under 5 years old in 1998 in Taiwan.^17^ Given these together, it indicates that incidence of KD in Chinese children was truly lower than that in Japanese children. However, the incidences in our study are higher than those reported in British Isles,^13^ Australia,^14^ Sweden,^21^ and Jamaica,^22^ but similar to figures in the recent reports in the United States.^10^^,^^11^ It should be noted that the prevalence of KD in Hawaii where live a high proportion of people of Asian ancestry is also different from that in Japan, which indicated that although genetic susceptibility may play an important role in the development of KD, other factors such as environment, foods, etc. may also contribute to the development of the disease as well.

The age distribution at onset of KD in our study is similar to the data of Beijing.^16^ The male predominance is similar to previous reports. However, the seasonal distribution in this study is similar to that in Beijing, but different from other reports. Our data showed that KD was more common in spring and summer. This was more obvious in girls, in whom the occurrence appeared to be higher in March, and reached the peak in July. For boys the higher peak was in May, and there was another small peak in September. The lowest occurrence was in February. However, as compared with Japanese data in which was shown more patients in Winter,^6^^-^^9^ and American data in which more patients was shown in Spring and Winter,^18^ it implicates that climate may relate to the susceptibility to KD. Bronstein et al^11^ had found that incidence of KD was inversely correlated with average monthly temperature and positively correlated with average monthly precipitation in San Diego. Because the data on seasonal variation in monthly rainfall and temperature was not obtained in our study, we could not demonstrate the relationship between climate status and susceptibility to KD. However, it is not consistent with the results of Bronstein et al because the summer season includes the hottest months in Shanghai. Previous studies have shown that respiratory virus infection predominated in the spring season and that enterovirus infection epidemics occurred in summer in both Shanghai and Beijing each year. This indicates that there might be some links between the seasonal changes of KD and the epidemics of viral infection. Similar to the increasing trend of incidence in Japan,^6^^,^^7^ the United States,^18^ Beijing,^19^ Taiwan,^17^ and England,^23^ the incidence of KD in Shanghai tends to increase from 1998 through 2002. The reasons remain unclear.

The clinical manifestations of KD in Shanghai are similar to those in previous reports.^6^^,^^7^^,^^12^^,^^14^^,^^19^ Fever lasting for 5 days or longer was the most common symptom, followed by oral changes and red lips, extremities desquamate, rashes, bilateral conjunctive congestion, cervical lymph node enlargement, and extremities swelling. Interestingly, crissum desquamate was found in 45.2% of the patients in this study, which was seldom mentioned in the previous reports.

Frequency of cardiac abnormalities in this study was 24.3%, which is similar to the previous reports from British Isles, Australia and Beijing.^13^^,^^14^^,^^19^ However, de Zorzi et al.^24^ reported that among patients with KD whose coronary arteries were classified as normal by the Japanese Ministry of Health criteria, body surface area-adjusted coronary dimensions were larger than expected in the acute, convalescent and late phases of the disease. Thus, coronary dilatation in KD might be more prevalent than previously reported. The cardiac lesions of KD were the most frequent under 1 year of age with the peak at 8.4 month in our data. The proportion of cardiac abnormalities was not different between boys (27.1%) and girls (22.4%). The occurrence rate of coronary artery lesions including dilatation and aneurysm was 19.8%, which were the most common cardiac abnormalities. Giant coronary aneurysm accounted for 15.8% of coronary aneurysm. In this study gamma-globulin therapy was given in 71.8% of patients. The dosage of 1000 mg/kg once or 2000 mg/kg once had been used more often than other dosage since 2000. Although our data could not prove the effects of administration of intravenous gamma-globulin on prevention of coronary artery lesions, there was a tendency that administration of intravenous gamma-globulin in the dosage of 1000mg or 2000mg/kg once may reduce the lesions. Coronary artery lesions occurred more frequently in the patients with hypoalbuminemia. It should be noted that patients who received corticosteroid treatment in our data more often had coronary artery lesions, which illustrates that it should be considered in every aspects before corticosteroid is used although it is difficult to assess the treatment based on this observational study.

The case-fatality rate of KD in our data was 0.26%. Rupture of coronary aneurysm and congestive heart failure at acute and subacute stage of the disease were the causes of death. A second onset of the disease occurred in 1.82% of patients in 1-35 months after the first onset, which was similar to previous reports in Japan. No specific factors were observed to be associated with the recurrence in this study.
